# A molecular beacon assay for monitoring RNA splicing

**DOI:** 10.1093/nar/gkac242

**Published:** 2022-04-19

**Authors:** Qusay Q Omran, Olga Fedorova, Tianshuo Liu, Anna M Pyle

**Affiliations:** Department of Chemistry, Yale University, New Haven, CT 06520, USA; Department of Molecular, Cellular and Developmental Biology Yale University, New Haven, CT 06520, USA; Howard Hughes Medical Institute, Yale University, New Haven, CT 06520, USA; Department of Molecular, Cellular and Developmental Biology Yale University, New Haven, CT 06520, USA; Department of Chemistry, Yale University, New Haven, CT 06520, USA; Department of Molecular, Cellular and Developmental Biology Yale University, New Haven, CT 06520, USA; Howard Hughes Medical Institute, Yale University, New Haven, CT 06520, USA

## Abstract

Small molecule targeting of self-splicing RNAs like group I and II introns has been limited in part by the lack of a universal high-throughput screening platform for studies of splicing inhibition and kinetics. Here, we present the development of a molecular beacon assay for monitoring the accumulation of spliced exons during RNA splicing reactions. In this case, we applied it to the autocatalyzed reaction of the H.c.LSU group II intron found in the mitochondria of the pathogenic dimorphic fungus *Histoplasma capsulatum*. We find that a molecular beacon with the loop length of 18 nucleotides selectively recognizes ligated exons formed during self-splicing and exhibits high fluorescent signal upon binding of its target. We demonstrate that the fluorescent assay using molecular beacons can be successfully applied to kinetic characterization of the splicing reaction and determination of inhibition constants for small molecules. The results presented herein offer support for a molecular beacon approach to identifying small molecule inhibitors of intron splicing.

## INTRODUCTION

**Figure 1.**
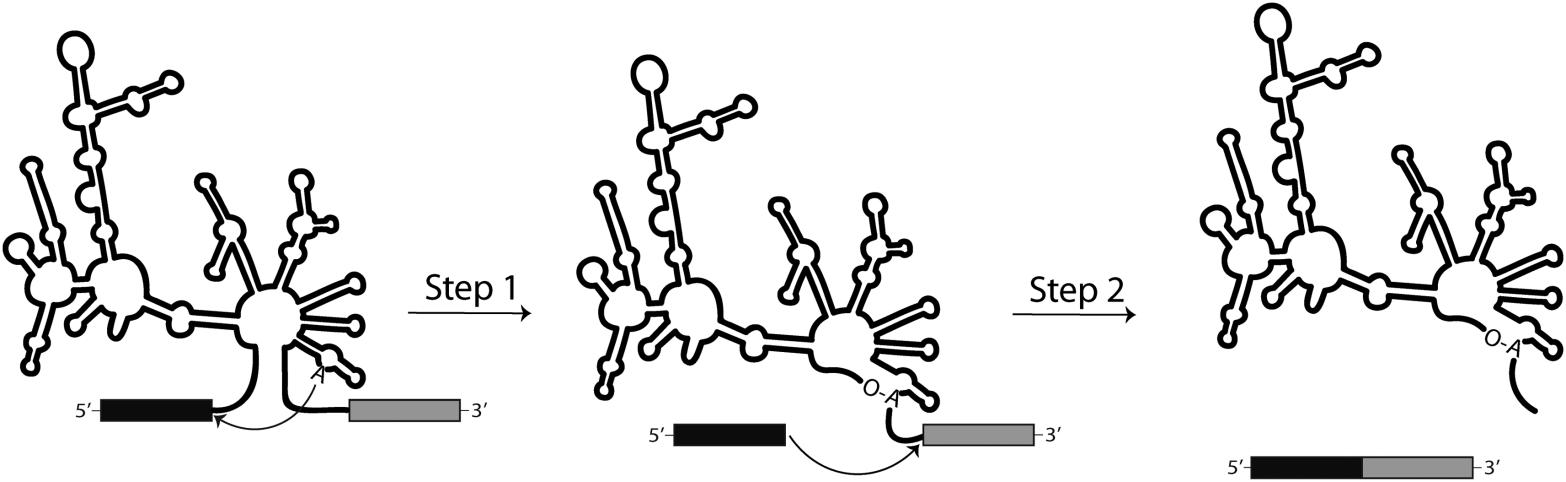
Schematic of the group II intron self-splicing reaction proceeding via the branching pathway. Black and grey rectangles represent 5′- and 3′-exons, respectively. Thin black line depicts the secondary structure schematic of the intron.

Group II introns are a class of autocatalytic ribozymes found in bacteria and the organellar genomes of fungi, plants and protists (1,2). These introns have long been thought to represent an ancient predecessor of the eukaryotic spliceosome, which shares the same characteristic two-step sequential splicing mechanism (Figure 1) (3–5). Despite a lack of phylogenetic conservation at the sequence level, group II introns have highly conserved secondary and tertiary structures (3,6). Because of their distinctive structure and ubiquity in mitochondrial genes responsible for the respiration of fungi, group II introns represent a promising therapeutic target that has largely been neglected by traditional screening and drug discovery platforms (7).

While small molecule targeting of RNA is a relatively new field, promising intron drug targets are being continually identified in human pathogens. One such intron, found in the ribosomal RNA (rRNA) from the large subunit (LSU) of the mitochondrial ribosome of *Histoplasma capsulatum* (Supplementary Figure S1), was recently identified by a bioinformatics approach and shown to exhibit self-splicing activity in the presence of catalytic Mg^2+^*in vitro* (8). Endemic to river valleys, *H. capsulatum* is a dimorphic fungus responsible for histoplasmosis, which is the most prevalent dimorphic fungal infection in the United States and a significantly underdiagnosed disease globally (9). The identified *H. capsulatum* group II intron from the mitochondrial ribosomal large subunit RNA (H.c.LSU) exhibits a relatively slower splicing rate constant relative to other group II introns, rendering it a useful self-splicing model for the development of a small molecule screening assay.

**Figure 2.**
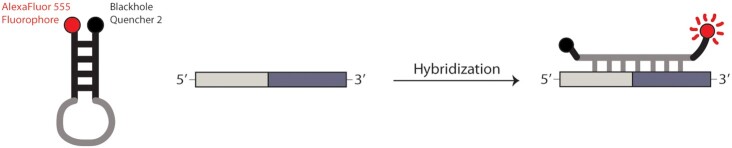
Schematic of molecular beacon hybridization to a target sequence. The target sequence is shown as a grey rectangle. The part of the molecular beacon complementary to the target sequence is shown in grey. The fluorophore in the molecular beacon stem-loop is shown in red and the quencher is in black.

Molecular beacons are fluorescent DNA-based probes that can detect specific sequences by complementary hybridization (10). To achieve the most sensitive detection of nucleic acid sequences, molecular beacons are often designed as stem-loop structures in which the fluorophore and quencher dyes are covalently attached at the termini of an oligonucleotide, in forced proximity with each other. Upon binding a complementary target sequence, however, the beacon will open and hybridize to the target, thereby increasing the distance between the fluorophore and the quencher and allowing fluorescence to serve as a signal for target identification in solution (Figure 2). Molecular beacons have become a powerful chemical biology tool, and they are commonly used to study RNA cellular localization and intronic structure (11,12). At least one high-throughput screening assay based on molecular beacons has been developed to identify modulators of RNA targets, such as miRNAs (13).

To our knowledge, however, it has not yet been demonstrated that RNA splicing can be monitored or interrogated using a molecular beacon approach. Here we demonstrate that molecular beacons can provide a reliable readout of RNA splicing kinetics and that the resulting methods are sufficiently generalizable and sensitive for application in high-throughput assays for identification of small molecule splicing inhibitors. Implementation of this assay for monitoring splicing and inhibition of the H.c.LSU group II intron provides a major step toward the creation of improved assays for small molecule targeting of self-splicing and pre-mRNA introns.

## MATERIALS AND METHODS

### *In vitro* transcription

Large-scale transcription of H.c.LSU group II intron precursor RNA (HC preRNA) was carried out as previously described (14) using the pLTS101 plasmid linearized with *BamH*I restriction enzyme. Radiolabeled HC preRNA was also prepared by *in vitro* transcription in the presence of ^32^P-α-UTP, as previously described (15). All RNAs were stored at −80°C in the RNA storage buffer (10 mM MOPS (pH 6.0), 1 mM EDTA).

### Molecular beacon and RNA target synthesis

RNA oligonucleotide HCSE (5′-ACUCUAGGUAGACGAGAAGACCCUAUGCAGCU-3′) was synthesized on a MerMade 12 DNA−RNA synthesizer (BioAutomation) using TBDMS RNA phosphoramidites (TxBio), deprotected and purified on an 18% denaturing polyacrylamide gel as previously described (16,17).

DNA oligonucleotides MB14 (5′-Am-CCAGGAGGGTCTTCTCGTCCCTGG-BHQ2-3′, loop sequence is underlined), MB18 (5′-Am-CCAGGATAGGGTCTTCTCGTCTACCTGG-BHQ2-3′, loop sequence is underlined) containing the 3′-terminal Black Hole Quencher 2 (BHQ2) and 5′-terminal aminomodifier C3 TFA (Am) (Glen Research) were synthesized on a MerMade 12 DNA–RNA synthesizer (BioAutomation) using UltraMild Base protection DNA phosphoramidites (Glen Research). The oligonucleotides were then deprotected using 28–30% ammonium hydroxide (J.T. Baker) at room temperature for 24 h and purified on an 18% denaturing polyacrylamide gel.

### Fluorescent labeling of molecular beacons

Purified MB14 and MB18 containing BHQ2 at the 3′-end and the aminomodifier C3-TFA at the 5′-end were covalently attached to the NHS ester of the AlexaFluor 555 dye (Life Technologies Corp.) via the primary amino group on the aminomodifier nucleotide. The beacons were dissolved in a 200 μl solution of 0.25 M NaHCO_3_ buffer (pH 9.2) before being combined with a 200 μl dimethylformamide solution containing 0.5 mg of the AlexaFluor 555 NHS ester dye. The labeling reaction was allowed to proceed at room temperature for 2 h, and the fluorescently labeled products were then purified on a 18% denaturing polyacrylamide gel and stored in the RNA storage buffer as above.

### Detection of the spliced exons using molecular beacons

Wells of black 96-well plates (Corning 3792) were filled with 50 μl of solution containing 100 nM HCSE or 200 nM *H.c.LSU*, 50 mM HEPES (pH 7.5), 150 mM NH_4_Cl, and 10 mM MgCl_2_ in water (optimal reaction conditions). Where indicated, 10 mM MgCl_2_ was omitted or 10 mM EDTA was added to the reaction mixture. The reaction mixture was incubated at 37°C for 2 h. Then, 50 nM of MB14 or MB18 was added to each well. The plate was then heated to 70°C for 5 min and incubated at 37°C for 30 min before analysis on a Synergy H1 plate reader (BioTek) using excitation wavelength of 540 nm and emission wavelength of 590 nm.

### Determination of self-splicing reaction rate constants

For each reaction, 10 wells of black 96-well plates (Corning 3792) (corresponding to 10 time points) were filled with 50 μl of a solution containing 200 nM HC preRNA (unlabeled or ^32^P-body labeled), 50 mM HEPES (pH 7.5), 150 mM NH_4_Cl and 10 mM MgCl_2_ in water. The reaction was allowed to run at 37°C. At each time point, 10 mM EDTA was added to the respective well in order to quench the splicing reaction. Once the time course was completed and all reaction wells were quenched, 50 nM MB18 was added to each well. The plate was then heated to 70°C for 5 min and allowed to cool to 37°C for 30 min before analysis on a Synergy H1 plate reader (BioTek). The GraphPad Prism software package was used to fit the self-splicing time course data to an exponential function that accounts for a time lag (Equation 1):

(1) $$\begin{equation*}{{A * }}{{\rm{e}}^{{{( - B(t - C))}}}}{{ + D}}\end{equation*}$$

where *A* represents the *y*-intercept, *B* was extracted as the reaction rate constant *k*_obs_, *C* was the time lag parameter and *D* represents a vertical shift parameter.

In order to compare the reaction rate constants determined using the radioanalytical and molecular beacon assays, ^32^-P body-labeled precursor RNA was added to the reaction, and 5 μl were withdrawn from each aliquot prior to MB18 addition, mixed with 5 μl of the denaturing loading buffer (80 mM Tris–HCl, pH 7.5, 8M urea, 0.05% each of xylene cyanol and bromophenol blue, 20% sucrose, 1.5 mM EDTA) and analyzed on a 5% denaturing polyacrylamide gel. Bands corresponding to the precursor and splicing products were visualized on an Amersham Typhoon phosphorimager and quantified using the ImageQuant TL imaging software package. The fraction of spliced exons was then plotted over time and fit to a simple exponential equation for reaction rate constant determination as described.

### Determination of the K_i_ for mitoxantrone inhibition of the self-splicing reaction

Since mitoxantrone as well as other compounds may partially quench fluorescence (18), it was important to set up the dose-response assay so that it would not be influenced by the quenching effect. Therefore, instead of taking single measurements at a specific mitoxantrone concentration, we carried out time courses of splicing reactions in the presence of varying concentrations of mitoxantrone (as described above). Using this setup, any quenching effect would be the same for all time points taken at a specific mitoxantrone concentration, and will therefore not affect the observed reaction rate. Reaction rate constants were determined in the presence of 14 different mitoxantrone concentrations, ranging from 5 nM to 300 μM, using MB18 as described above. The derived reaction rate constants were plotted against the respective inhibitor concentrations, and the GraphPad Prism software package was used to fit the data to a four-parameter logistic function (Equation 2):

(2) $$\begin{equation*}{{A}}\,{\rm{ + }}\,\left( {{{B}}\,{\rm{ - }}\,{{A}}} \right){\rm{/}}\left( {{\rm{1 + }}{{\left( {{{x/C}}} \right)}^{{D}}}} \right)\end{equation*}$$

where *A* is the minimum response, *B* is the maximum response, *C* is the *K*_I_ and *D* is the slope parameter.

## RESULTS AND DISCUSSION

### Design of molecular beacons targeting spliced exons

Based on the principles of molecular beacon design and our understanding of the H.c.LSU group II intron, we chose to synthesize two DNA beacons designed to hybridize specifically to the junction formed when the 5′- and 3′-exons are ligated together during the second step of the sequential self-splicing reaction (Figures 1, 2) (19,20). The stem length of both beacons was kept constant at five nucleotides, in keeping with earlier findings that this length ensured low background fluorescence without compromising the rate of hybridization with the target (12). The length of the loop portion complementary to the ligated spliced exons was varied, however, in order to test the activity and specificity dependence of the designed beacons. While beacons with longer loops form a longer and more stable set of base pairs with their target sequence, this greater length also risks decreasing their binding specificity, as longer beacons are more likely to hybridize with off-target sequences that share some identity with the target (19). Taking these considerations into account, the shorter beacon was designed with a loop length of 14 nucleotides (MB14), while the longer beacon had a loop length of 18 nucleotides (MB18) (see Materials and Methods). To aid in optimizing the beacon assay and to serve as a positive control for beacon hybridization to the spliced exons, a synthetic 32-nucleotide RNA oligo (HCSE) with the same sequence identity as the spliced exons junction was also prepared.

### Molecular beacons selectively recognize ligated exon products

**Figure 3.**
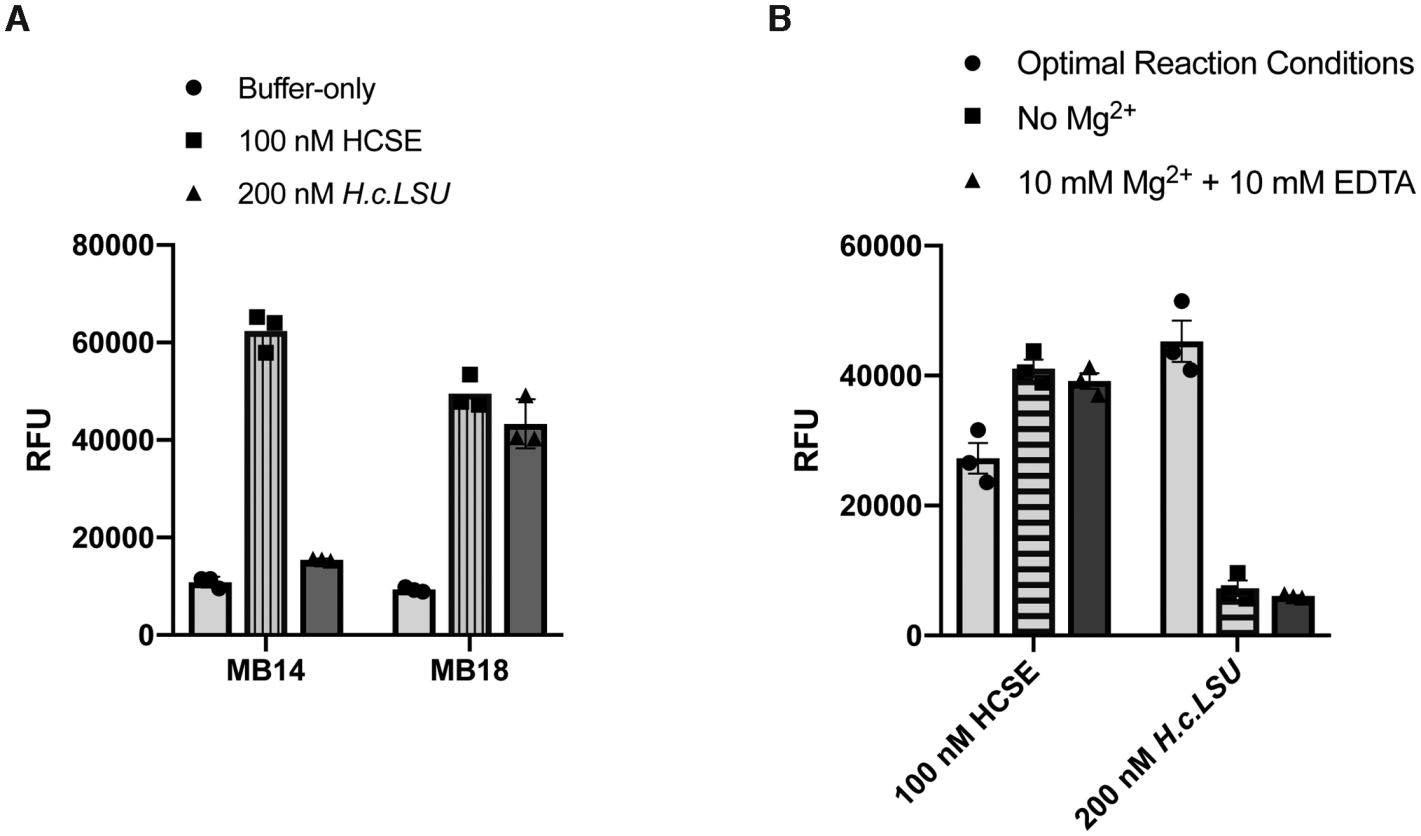
(**A**) Recognition of the synthetic target RNA and the ligated exons formed during splicing by molecular beacons. Beacons were incubated with buffer-only (negative control, light grey bars), with the HC preRNA splicing reaction (dark grey bars)), or with the HCSE spliced exon oligonucleotide (positive control, striped bars). Buffer composition in each well reflects optimal reaction conditions. Y-axis represents relative fluorescent units (RFU). Data represent the average of *n* = 3 independent experiments. Error bars are s.e.m. (**B**) Comparison of the molecular beacon signal under different reaction conditions. 50 nM MB18 was incubated with either the synthetic HCSE oligo or the HC preRNA in either optimal reaction buffer (light grey bars), buffer lacking MgCl_2_ (striped bars), or in reaction buffer containing an equimolar amount of EDTA (dark grey bars). Y-axis represents relative fluorescent units (RFU). Data represent the average of *n* = 3 independent experiments. Error bars are s.e.m.

To confirm that the designed beacons behave as expected in the presence of the target sequence (spliced exons), the activities of MB14 and MB18 at 50 nM were assayed in the presence of 100 nM HCSE target oligonucleotide (Figure 3A), which has the same sequence as spliced exons. Notably, both beacons exhibited high fluorescent signal upon binding the HCSE target sequence.

We then tested whether the beacons could detect ligated exons formed during the course of the corresponding intron self-splicing reaction. The reaction was carried out under the optimal conditions for splicing via branching (see Materials and methods).

The HC preRNA was studied at 200 nM to ensure sufficient accumulation of ligated exons such that we would expect at least a 5-fold signal to noise ratio upon hybridization with the beacon. After the splicing reaction was carried out for two hours, 50 nM of beacon was added and the reaction mixture was denatured at 70°C for 5 min. Then the samples were incubated at 37°C for 30 min to allow the beacon to hybridize to the target. We found that, under these conditions, MB18 produced strong fluorescent signal upon binding the ligated exons that are generated during the splicing reaction, but the signal for MB14 was much weaker (Figure 3A). Based on these results, MB18 was chosen for further experiments.

Since MB18 can potentially hybridize to the precursor RNA by binding to the last nine nucleotides of the 5′-exon and then reaching across the intron to hybridize with first nine nucleotides of the 3′-exon, it was important to test its ability to differentiate between the precursor RNA and the ligated exons. For this purpose, we carried out a variation of the experiment described above in which the mixture contained no Mg^2+^ ions, thereby precluding self-splicing of precursor RNA. However, the reaction contained monovalent ions (150 mM NH_4_Cl), which are sufficient for promoting RNA and DNA duplex formation. In the absence of Mg^2+^, solutions containing the HC preRNA and MB18 displayed only background fluorescence, at a level comparable to that of samples that lacked spliced exon sequences (Figure 3B, right). At the same time, the absence of Mg^2+^ ions did not affect the ability of the MB18 to hybridize with the synthetic RNA target oligonucleotide identical to the spliced exons, (Figure 3B, left). These data indicate that MB18 selectively binds to the ligated exons and not to the precursor RNA.

EDTA is commonly used to quench splicing reactions because it sequesters the magnesium ions required for splicing catalysis. When 10 mM EDTA is added together with 10 mM Mg^2+^ prior to incubation, a solution containing MB18 beacon and the HC preRNA displays only background fluorescence, as if Mg^2+^ were not present in the solution (Figure 3B, right). This control demonstrates that EDTA does not impede hybridization of the beacon to the target, evidenced by similar levels of activity towards the HCSE oligo in wells with or without the sequestering agent (Figure 3B, left), but it does quench the splicing reaction. These data indicate that equimolar amounts of EDTA relative to Mg^2+^ provide an effective quench of the splicing reaction.

### Application of molecular beacons for monitoring splicing kinetics

**Figure 4.**
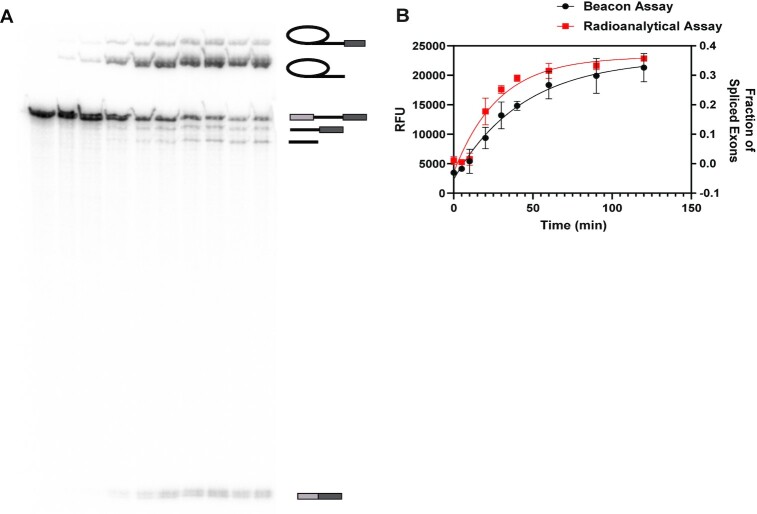
Parallel analysis of self-splicing kinetics by molecular beacon and radioanalytical methods. (**A**) Representative 5% denaturing polyacrylamide gel of the radiolabeled HC preRNA showing depletion of precursor and accumulation of reaction products, including spliced exons, over the course of the reaction. Schematics on the right indicate splicing products (top to bottom): lariat intron-3′-exon intermediate, lariat intron, precursor RNA, linear intron-3′-exon intermediate, linear intron, spliced exons. (**B**) Accumulation of ligated exons over time, monitored by radioanalytical (red) and molecular beacon (black) assays. Data were fit to a single exponential equation to determine reaction rate constants (0.023 ± 0.009 min^−1^ from the beacon assay and 0.036 ± 0.009 min^−1^ from the radioanalytical assay). Data represent the average of *n* = 3 independent experiments. Error bars are s.e.m.

Kinetic characterization of self-splicing is typically performed using radioanalytical methods, in which the precursor RNA is internally labelled with a ^32^P-α-NTP and progress of the splicing reaction is monitored over time by electrophoretic separation of reaction products. In order to determine if accumulation of spliced exons can be accurately measured using molecular beacons, we monitored the same splicing reaction using both the radioanalytical and molecular beacon fluorescent methods. In this experiment, ten reaction chambers (plate wells) were filled with a solution of 200 nM of cold HC preRNA, which had been spiked with 1 nM of [α-^32^P]-UTP body-labeled HC preRNA, and the splicing reaction was initiated by adding a solution of MgCl_2_ (10 mM final concentration). Subsequently, the reaction in each well was quenched at specified time points by addition of 10 mM EDTA. The contents of each well were then split and analyzed with the two analytic methods in parallel. For the radioanalytical splicing assay, 5 μl of solution from a given well were withdrawn, mixed with the denaturing loading buffer and the products were separated and visualized on a 5% denaturing polyacrylamide gel as described in Materials and Methods (Figure 4A, B). For the MB18 assay, the remaining contents of each well (45 μl) were combined with 5 μl of a MB18 beacon solution (50 nM final concentration), beacon was allowed to anneal, and the results were analyzed on a plate reader, as described in Materials and Methods (Figure 4B). When data from the two methods were compared, we observed changes in MB18 fluorescent signal that correlated directly with the accumulation of radiolabeled spliced exons (Figure 4B), ultimately resulting in similar rate constants (0.036 ± 0.009 min^−1^ for radioanalytical and 0.023 ± 0.009 min^−1^ for the beacon assay). These data indicate that the molecular beacon assay can be used to accurately monitor the splicing of RNA precursors.

Given that the testing of small molecule inhibitors often involves dissolution of compounds in DMSO, it was important to test the impact of 5% dimethyl sulfoxide (DMSO) on the progression of the splicing reaction and the ability of MB18 to detect spliced exon product. We observe that addition of 5% DMSO to the reaction had no deleterious effect on detection and actually increased the signal-to-background ratio of the beacon assay, resulting in a ∼6-fold window for a 90 min time course (Supplementary Figure S2). That 5% DMSO improves activity of the MB18 beacon is consistent with previous studies of molecular beacon fluorescence activity in the presence of organic solvents, where it has been shown that solvents like DMSO decrease the activation energy required for the strand hybridization reaction (21). It is notable that DMSO has no significant impact on the splicing reaction kinetics, as we observe reaction rates of 0.043 ± 0.013 min^−1^ and 0.048 ± 0.012 min^−1^ in the presence and absence of DMSO, respectively. These findings establish the utility of the beacon method for high-throughput screening of and kinetic characterization of small molecule splicing inhibitors.

### Determination of a small molecule inhibition constant using the molecular beacon assay

**Figure 5.**
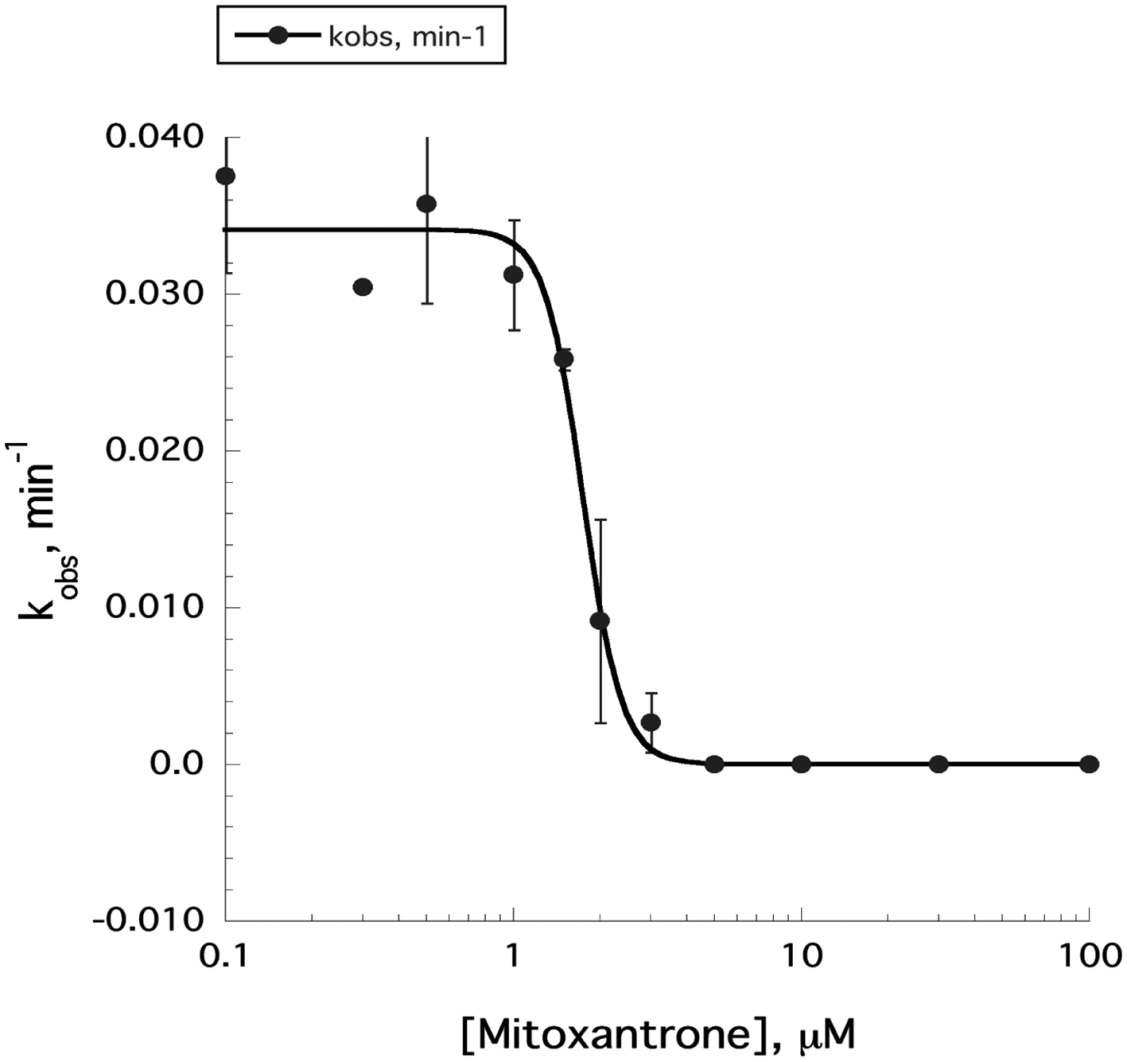
Plot of splicing rate constant (*k*_obs_) as a function of mitoxantrone concentrations. Each data point represents the rate constant determined by fitting a single exponential curve to a time course of the self-splicing reaction in the presence of the respective inhibitor concentration. A logistic curve fit produced a *K*_I_ value of 1.79 ± 0.32 μM. Data represent the average of *n* = 2 independent experiments. Error bars are s.e.m.

Given that the molecular beacon assay can be used to accurately determine splicing rate constants, we set out to determine if the assay can be used to measure small molecule inhibition of splicing under conditions amenable to high throughput analysis. Since there are no known specific inhibitors of H.C. LSU group II introns, we tested inhibition with the non-specific RNA binder mitoxantrone (Figure 5). Originally discovered as a strong binder of stem loop RNAs (22,23), mitoxantrone is also known to inhibit splicing reactions (8), making it a useful tool compound for K_I_ determination using our novel beacon assay. In addition, mitoxantrone is already known to inhibit splicing of the H.C. LSU group II intron (8).

To test the effect of mitoxantrone, we used MB18 to monitor the efficiency of splicing in the presence of increasing drug concentrations. Reaction rate constants were plotted against the concentration of the inhibitor to yield an inhibition constant of 1.79 ± 0.32 μM (Figure 5, Supplementary Figure S3), indicating strong inhibition of the splicing reaction. This is consistent with previously published results where the mitoxantrone inhibition constant for the self-splicing of the H.C. LSU group II intron was measured using a radioanalytical method and reported as 0.64 ± 0.08 μM (8). More importantly, this experiment demonstrates that the molecular beacon platform allows for construction of a typical dose–response curve, with characteristic plateaus and a sloped regions needed for quantitative analysis.

### Conclusion and perspectives

This study focuses on the design and application of molecular beacons that are sensitive to the second and final step of RNA splicing (exon ligation), although the assay can be easily adapted for use in other systems. For example, it is also possible to design beacons for monitoring formation of the lariat-3′-exon intermediates or 5′-exon-intron junctions, which would facilitate analysis of the first step of self-splicing. Though only one beacon is necessary for high-throughput screening purposes, synthesis of alternative beacons could enable independent determinations of individual reaction rates in a multiplexed assay.

While this particular beacon assay was optimized for monitoring self-splicing of group II introns, beacon assays for high-throughput screening and drug development can be readily designed for monitoring group I intron splicing (also common in fungal pathogens) and nuclear pre-mRNA splicing in metazoans (central to expression of specific genes). The design of beacons in other systems will necessitate careful consideration of the particular characteristics of a target intron's structure, exon sequences and self-splicing reaction mechanism. For example, the *Saccharomyces cerevisiae* ai5γ mitochondrial group II intron exhibits hydrolytic reopening of its spliced exons under certain reaction conditions (24). A molecular beacon designed for hybridization to the spliced exons of the ai5γ intron might therefore show relatively weak activity, while a design that instead focuses on formation of spliced lariat intron product could prove more effective. This limitation is not a concern for group I or pre-mRNA introns, which do not generally undergo reopening of their spliced exons product but which by contrast might not form lariat splicing products (25). For such systems, recognition of spliced exons by molecular beacons would be most ideal. The adaptability of the molecular beacon platform for monitoring various types of RNA splicing under high-throughput conditions will facilitate the screening of small molecules that target specific splicing reactions and isoforms.

## DATA AVAILABILITY

All relevant data are included in the paper and/or its supplementary information files.

## Supplementary Material

gkac242_Supplemental_File
