# Supplementary material for: A molecular beacon assay for monitoring RNA splicing
Source: Nucleic Acids Res. 2022 Apr 19;50(13):e74. doi: 10.1093/nar/gkac242 (PMC9303364; doi:10.1093/nar/gkac242)
Supplement: gkac242_Supplemental_File [file gkac242_supplemental_file.docx]

Supplementary information


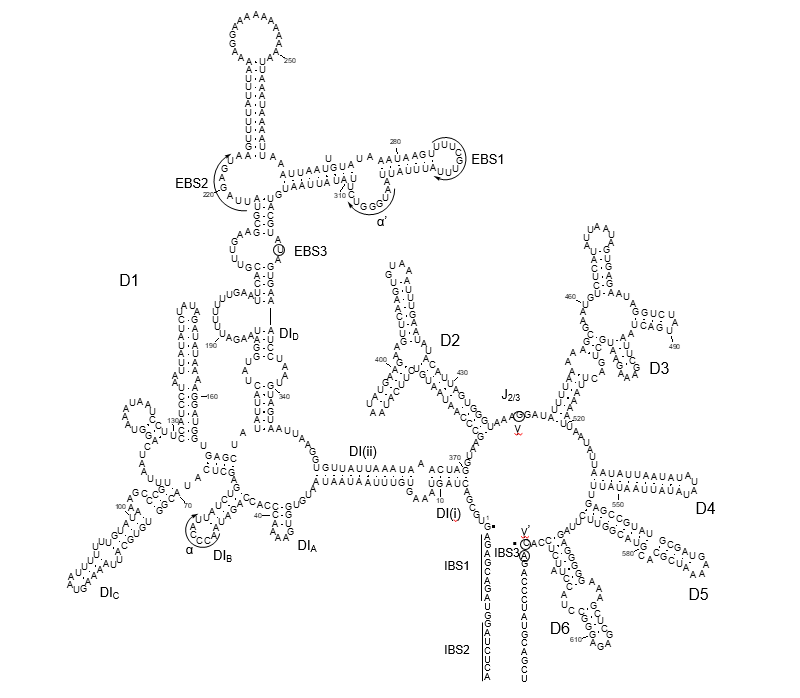


Supplementary Figure S1. Predicted secondary structure of the H.c.LSU group II intron. The conserved six domains observed for group II introns are labeled as D1-D6.


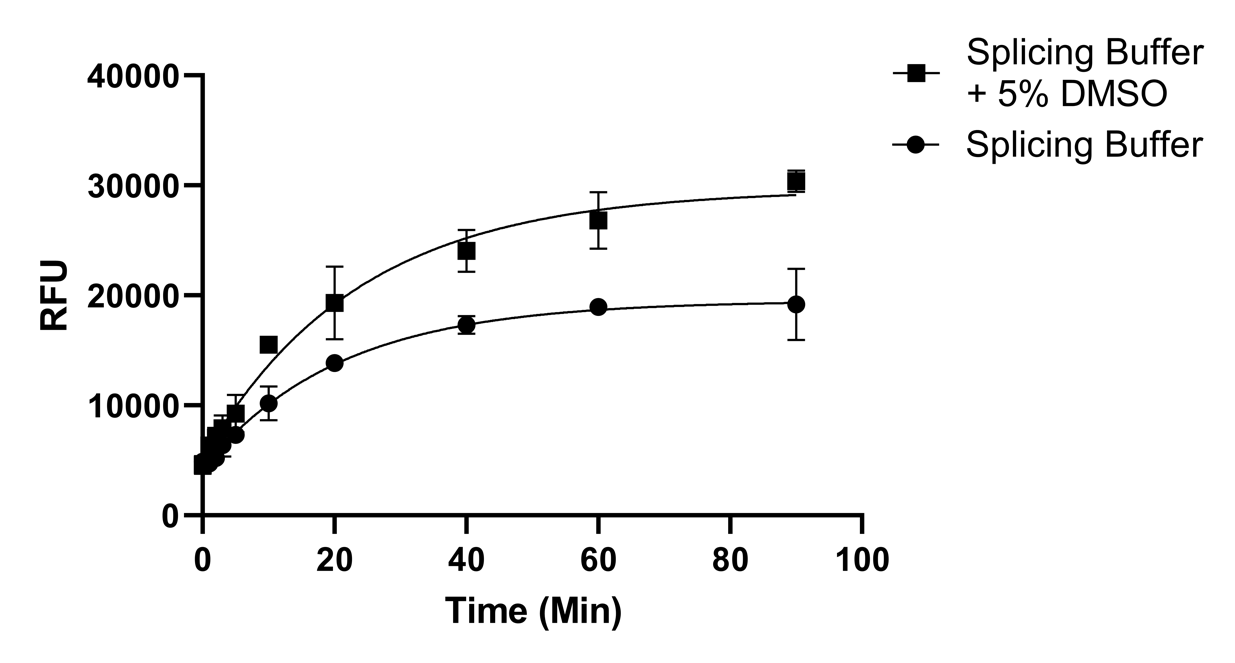


Supplementary Figure S2. Comparison of time courses of the H.c.LSU splicing reaction under optimal conditions (50 mM HEPES (pH 7.5), 150 mM NH_4_Cl, and 10 mM MgCl_2_) in the presence and in the absence of 5% DMSO. Each reaction was initiated with the addition of 10 mM MgCl_2_ and quenched at each timepoint with equimolar EDTA. Rate constants of 0.043 ± 0.013 min^-1^ and 0.048 ± 0.012 min^-1^ were derived for the reactions with and without 5% DMSO, respectively. Data represent the average of n = 2 independent experiments. Error bars are s.e.m.


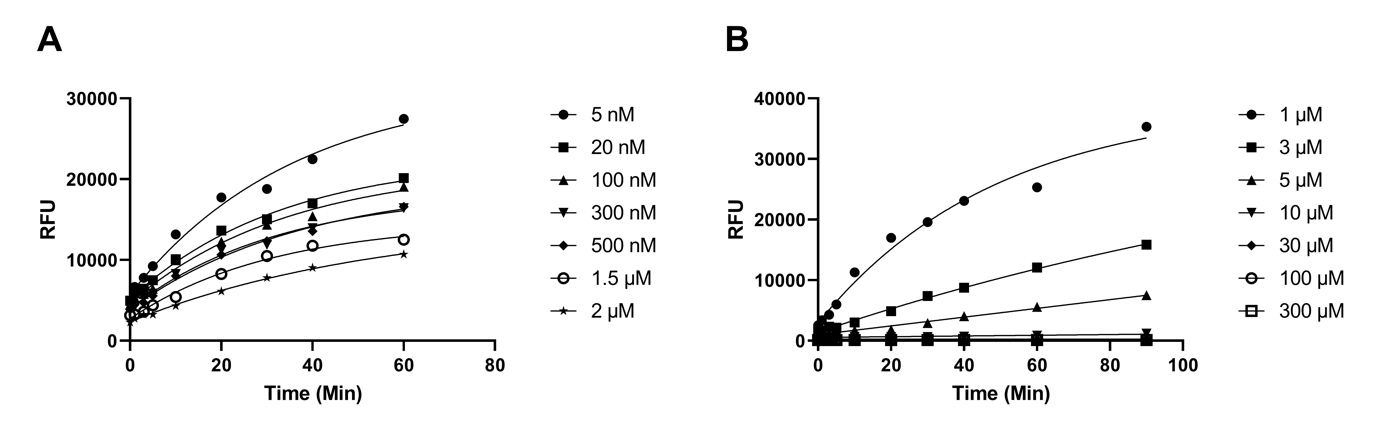


Supplementary Figure S3. Representative time course data for the determination of the Ki of mitoxantrone. Time courses of the splicing reaction in the presence of 5 nM to 2 μM mitoxantrone are shown in panel (A), while panel (B) shows the corresponding data for 1 μM to 300 μM mitoxantrone.
